# Epidermal stem cells (ESCs) accelerate diabetic wound healing via the Notch signalling pathway

**DOI:** 10.1042/BSR20160034

**Published:** 2016-08-05

**Authors:** Rong-Hua Yang, Shao-Hai Qi, Bin Shu, Shu-Bin Ruan, Ze-Peng Lin, Yan Lin, Rui Shen, Feng-Gang Zhang, Xiao-Dong Chen, Ju-Lin Xie

**Affiliations:** * Department of Burn Surgery, The First People's Hospital of Foshan, Foshan, Guangdong, China; † Department of Burn Surgery, First Affiliated Hospital of Sun Yat-Sen University, Guangzhou, Guangdong, China

**Keywords:** diabetic wound healing, epidermal stem cells (ESCs), Jagged1 (Jag1), migration, Notch signalling

## Abstract

Chronic, non-healing wounds are a major complication of diabetes. Recently, various cell therapies have been reported for promotion of diabetic wound healing. Epidermal stem cells (ESCs) are considered a powerful tool for tissue therapy. However, the effect and the mechanism of the therapeutic properties of ESCs in the diabetic wound healing are unclear. Herein, to determine the ability of ESCs to diabetic wound healing, a dorsal skin defect in a streptozotocin (STZ)-induced diabetes mellitus (DM) mouse model was used. ESCs were isolated from mouse skin. We found that both the mRNA and protein levels of a Notch ligand Jagged1 (Jag1), *Notch1* and Notch target gene Hairy Enhancer of Split-1 (Hes1) were significantly increased at the wound margins. In addition, we observed that Jag1 was high expressed in ESCs. Overexpression of Jag1 promotes ESCs migration, whereas knockdown Jag1 resulted in a significant reduction in ESCs migration *in vitro*. Importantly, Jag1 overexpression improves diabetic wound healing *in vivo*. These results provide evidence that ESCs accelerate diabetic wound healing via the Notch signalling pathway, and provide a promising potential for activation of the Notch pathway for the treatment of diabetic wound.

## Abstract (precis)

Epidermal stem cells (ESCs) accelerate diabetic wound healing via the Notch signalling pathway.

## INTRODUCTION

Diabetic skin ulcers are non-healing, chronic wounds that pose a major health burden to society [1]. Up to a quarter of diabetic patients will develop these ulcers in their lifetime and as a result, nearly one-fifth of these diabetic patients will require non-traumatic lower limb amputations [2]. Numerous advanced treatment options for the management of diabetic ulcers have been explored, including bioengineered skin substitutes, hyperbaric oxygen therapy and negative pressure dressings [3–5]. However, the effective therapies are still lacking.

Recently, stem cells appear to emerge as a promising wound healing therapy [6]. Such as mesenchymal stem cells (MSCs) [7–10], endothelial progenitor cells [11] and adipose-derived stromal cells [12] have been reported for the cell therapies of diabetic delayed wounds *in vivo*. As a practical therapeutic approach, skin has been considered most recently as a potential adult stem cell source. It is highly accessible, and autologous tissue can be obtained easily with minimal donor site complications. Moreover, skin is an abundant pluripotent and multipotent cell source with an immune privilege and the potential for self-replication [13,14]. Epidermal stem cells (ESCs) have been reported as a stem cell source for the treatment of diabetic wounds [15]. Moreover, ESCs are primitive, unique, multipotent stem cells. Apart from their multilineage differentiation ability, ESCs have inherent host compatibility, immunosuppressive ability, susceptibility to gene modification and extensive capacity for *in vitro* expansion [16,17]. However, the effect and the mechanism of the therapeutic properties of ESCs in the diabetic wound healing are largely unknown.

Notch signalling pathways play key role in cell-fate decision and differentiation in many tissues during embryonic and postnatal development [18]. Four mammalian Notch receptors have been identified, designated as Notch1 to Notch4. Interaction of Notch receptors with membrane-bound ligands of the Delta and Jagged families [Delta-like1 (Dll-1), Delta-like4 (Dll-4), Jagged1 (Jag1) and Jagged2 (Jag2)] induces γ-secretase-mediated cleavage and translocation of Notch intracellular domain (ICD) into the nucleus, where it interacts with the transcription factor C-promoter binding factor 1 (CBF-1), Suppressor of hairless (Su(H)), Caenorhabditis elegans (Lag-1) (CSL). Once bound to CSL, Notch ICD recruits other co-activators, including mastermind proteins, and this transcriptional activation complex induces the expression of downstream target genes, such as Hairy Enhancer of Split-1 (Hes1) [19]. Given identification of Notch signalling in skin, the application of the pathway may be a potential avenue to improve wound healing. The underlying molecular mechanisms for Notch signalling pathway related to wound repair is not clear.

In the present study, we aimed to evaluate the therapeutic properties of ESCs in the diabetic wound healing. Further, we investigated the role of Notch signalling pathway that controls the migration of ESCs involved in wound healing *in vitro* and *in vivo*.

## MATERIALS AND METHODS

### Animals and diabetic model

All procedures and experiments involving animals in the present study were performed in accordance with the National Institutes of Health Guide for Care and Use of Laboratory Animals (NIH Publication No. 86-23, Revised 1985). The study protocol was approved by the Animal Ethics Committee at The First People's Hospital of Foshan, China. Experimental mice (8-week-old C57BL/6 female mice) were purchased from Shanghai SLAC Laboratory Animal and were housed in the Animal Resource Facility. It has been reported that the mice receiving single streptozotocin (STZ) intraperitoneal injection at 150 and 200 mg/kg showed significantly decreased body weight and increased blood glucose. STZ injection at 200 mg/kg resulted in a significantly higher mortality rate and shorter survival time than STZ at 150 mg/kg (*P*<0.05). Intraperitoneal injection of STZ at 150 mg/kg is associated with a low mortality rate, a high successful modelling rate of diabetes and a long survival time in mice [20]. To avoid lethality and significant loss of body weight, the mice (*n*=30) were rendered diabetic by a single intraperitoneal injection of STZ (150 mg/kg; Sigma–Aldrich); mice in the control group (*n*=10) were injected with vehicle alone (0.01 M citrate buffer, pH 4.5). Mice were considered diabetic if plasma glucose levels >300 mg/dl 1 week after STZ injection. Mice with a successful course after diabetes induction subsequently were allocated to diabetic wound healing study.

### Animal study design

All experiments used a diabetic wound healing model developed and described previously and recently [15,21]. Briefly, animals were anesthetized, shaved and prepared according to standard sterile procedure. Two wounds (8 mm in diameter, 3–4 mm apart) were made on the back of each mouse by excising the skin and underlying panniculus carnosus. The animals were then divided into three experimental groups (*n*=5 per group): group 1, 100 μl of PBS application as control group; 1.0×10^6^ cells of ESCs suspended with 100 μl of PBS application as ESCs-treated group; 1.0×10^6^ cells of Lv-Jag1-ESCs suspended with 100 μl of PBS application as ESCs-treated group. Cultured ESCs or Lv-Jag1-ESCs were injected into the subcutaneous layer around the dorsal wounds. Digital photographs were taken at days 0, 5, 10, 15, 20, 28 and beginning on the day of wounding. Photographs were acquired with a 10-megapixel digital camera (Canon) from a distance of 5.0 cm, with the lens parallel to the wound. Time-to-closure was defined as the number of days for complete re-epithelialization. Wound area was measured using digital selection by the public domain software ImageJ (NIH). Percentage wound closure was calculated as {1 − [(wound area)/(original wound area)]} × 100%. At the end of the experiment, wounds were excised with 2 mm margin beyond the wound edge. Each sample was placed in optimal cutting temperature medium and processed for frozen sections.

### Isolation of ESCs

Skin samples from the back of 8-week-old C57BL/6 female mice were carefully and separately dissected free from other tissue, placed in Hank's balanced salt solution (HBSS), and cut into approximately 1 mm^2^ pieces using dissecting scissors. Then, the segments were digested in 0.25% trypsin/EDTA at 37°C for 45 min. The resulting cell suspensions were seeded and cultured in a six-well plate in Dulbecco's Modified Eagle Medium: Nutrient Mixture F-12 (DMEM/F12) medium (Gibco, Invitrogen) containing 15% embryonic stem cell screened FBS (ES-FBS; Gibco), 1% glutamine (Gibco), 1% penicillin–streptomycin (Gibco) and fibroblast growth factor-basic (bFGF) (Invitrogen, 4 ng/ml) at 37°C in 5% CO_2_ in humidified air. Cells were passaged every 4–6 days.

### Flow cytometry

The expression of ecto-5'-nucleotidase, cluster of differentiation 73 (CD73), cluster of differentiation 14 (CD14), Hematopoietic progenitor cell antigen (CD34) and protein tyrosine phosphatase, receptor type, C (CD45) were evaluated on ESCs obtained from mouse skin. Cells (1×10^6^) were suspended in 2% BSA/PBS and labelled with CD73, CD14, CD34 and CD45 (all from BD). Flow cytometry was performed using a FC500 flow cytometer (Beckman Coulter) and analysed by Beckman Coulter CXP software.

### Differentiation of ESCs

ESCs were cultured in StemXVivo MSC expansion media (R&D Systems) and differentiation was induced as indicated using the media supplements included in the mouse MSC functional identification kit (R&D Systems). Markers of osteocyte and chondrocyte lineages were detected using a sheep anti-mouse osteocalcin polyclonal antibody and a sheep anti-mouse collagen II antigen affinity purified polyclonal antibody respectively. In addition, the frozen sections were prepared to do the Oil Red O for lipid staining (Sigma–Aldrich).

### Quantitative real-time PCR

Total RNA was extracted from the cells or tissues by Trizol Reagent (Invitrogen). For mRNA detection, reverse transcribed cDNA was synthesized with the PrimeScript RT reagent Kit (TaKaRa) and quantitative real-time PCR (qRT-PCR) was performed with an asymmetrical cyanine dye (SYBR) Premix ExTaq (TaKaRa) with the Stratagene Mx3000P real-time PCR system (Agilent Technologies). β-Actin was used as internal controls for mRNA quantification. The relative expression ratio of mRNA was calculated by the 2^−ΔΔ^*^C^*_T_ method. PCR reactions for each gene were repeated three times. Independent experiments were done in triplicate. All the sequences of the PCR primers used in the present study are shown in Supplementary Table S1.

### Establishment of ESCs with stable expression of Jag1

Lentiviral vectors which overexpress Jag1 were purchased from GeneChem. A lentiviral vector expressing scrambled RNA was used as the control. ESCs were infected with lentiviral vector and polyclonal cells with GFP signals (over 80% of transfected cells) were selected for further experiments using FACS flow cytometry. Total RNA from these cell clones was isolated, and levels of ESCs were quantified using qRT-PCR.

### Transient transfection with siRNAs

siRNA for Jag1 was designed and synthesized by Guangzhou RiboBio. The sequence of the negative control (NC) was also designed by RiboBio. Twelve hours prior to transfection, cells were plated on to a six-well plate (Nest Biotech) at 30–50% confluence. TurboFect siRNA Transfection Reagent (Fermentas) was then used to transfect siRNA into cells according to the manufacturer's protocol. Cells were collected after 48–72 h for further experiments.

### Cell migration assay

For the cell migration assay, 1×10^4^ cells in 100 μl medium without FBS were seeded on a fibronectin-coated polycarbonate membrane insert in a transwell apparatus (Costar). In the lower chamber, 500 μl medium with 10% FBS was added as chemoattractant. After the cells were incubated for 6 h at 37°C in a 5% CO_2_ atmosphere, the insert was washed with PBS, and cells on the top surface of the insert were removed with a cotton swab. Cells adhering to the lower surface were fixed with methanol, stained and counted under a microscope in five predetermined fields (×100). All assays were independently repeated at least three times.

### Western blot analysis

The protein extracts from cells or tissues were separated in 12% SDS/PAGE gels and blotted on nitrocellulose membranes, and probed with specific antibodies. The primary antibodies against Jag1, Notch1, Hes1 and β-actin were purchased from Cell Signaling Technology. After incubation with primary antibodies, the membranes were washed with TBS/0.05% Tween-20 and incubated with horseradish-peroxidase-conjugated secondary antibodies at room temperature for 1 h. Signals were detected using enhanced chemiluminescence reagents (Pierce).

### Immunohistochemistry staining

Skin tissue samples were routinely fixed with formalin and embedded in paraffin. The paraffin-embedded fixed tissue sections (4 μm thick) were deparaffinized and rehydrated. Following rehydration, antigen retrieval was carried out by placing the slides in 10 mmol/l sodium citrate buffer (pH 6.0) at 95°C for 20 min followed by 20 min cooling. The sections were then washed in PBS and non-specific binding sites were blocked with 1% BSA with 2% goat serum in PBS before incubation with specific antibody. The primary antibodies against CD73 were purchased from Cell Signaling Technology. After washing, the sections were incubated with biotinylated secondary antibody followed by horseradish-peroxidase-conjugated streptavidin. The sections were further incubated with 2,4-diaminobenzidine substrate and counterstained with haematoxylin.

### Statistical analysis

Data are presented as mean ± S.D. unless otherwise indicated. The statistical significance of the difference between the values of control and treatment groups was determined by Student's *t* test using Prism version 5 (GraphPad Software). Values of *P*<0.05 were considered statistically significant.

## RESULTS

### Characterization and differentiation of ESCs *in vitro*

**Figure 1:**
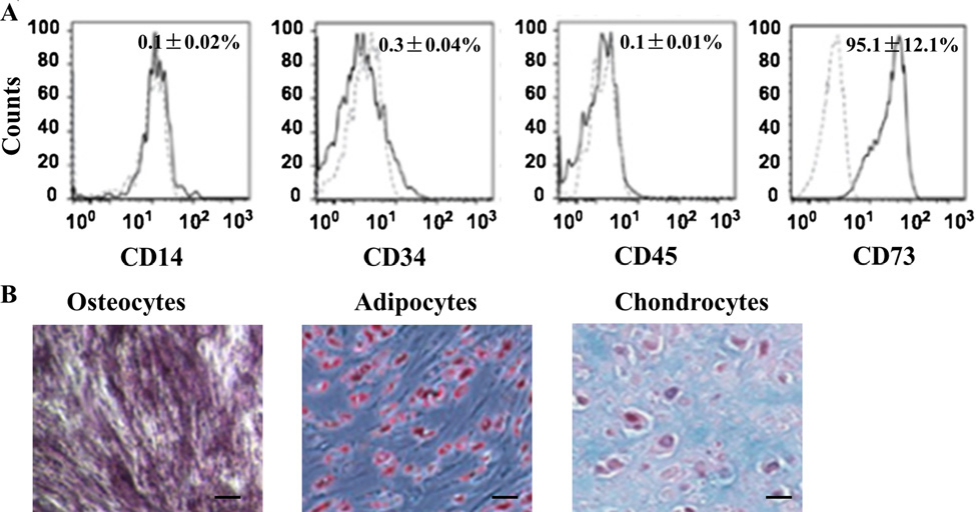
The characterization and differentiation of ESCs (**A**) Phenotype of ESCs by flow cytometry. ESCs were expanded in the culture and demonstrated positive surface staining for CD73, but not for CD45, CD34 or CD14. (**B**) ESCs were able to differentiate into osteoblasts, adipocytes (Oil Red O staining) and chondroblasts under standard *in vitro* differentiation conditions; Scale bars, 100 μm.

The cells presumed to be ESCs derived from mice skin were harvested and isolated for ESC culture. Flow cytometry was used to detect the phenotype of the ESCs. The purity of ESCs preparations was >95%, as judged by positive surface staining for CD73 (95.1%), and lack of expression of CD14, CD34 and CD45 (Figure 1A). Moreover, ESCs were multipotent, as determined by their ability to differentiate into osteoblasts, adipocytes and chondroblasts (Figure 1B). These results indicated that mouse skin-derived cells have the characteristics of ESCs.

### High expression of Jag1 in diabetic wound skin tissues was associated with activation of the Notch pathway

**Figure 2:**
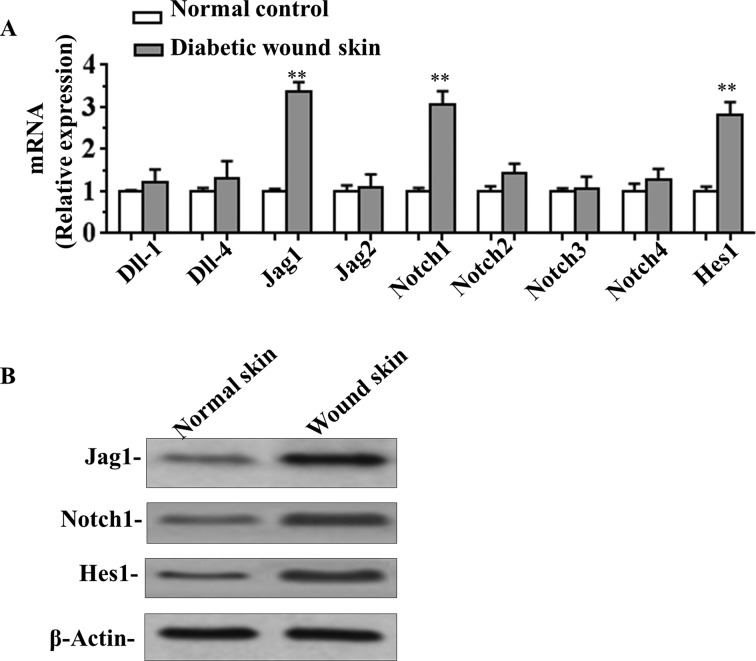
The expression of Notch receptors and ligands in the diabetic wound skin (**A**) mRNA levels of four Notch ligands (*Dll-1*, *Dll-4*, *Jag1* and *Jag2*), four Notch receptors (*Notch1–4*) and *Hes1* in normal or diabetic wound skin tissues were analysed by qRT-PCR. (**B**) Western blot assay of Jag1, Notch1 and Hes1 expression in normal or diabetic wound skin. Data are presented as the mean ± S.D. from three independent experiments; **P<0.01 compared with the control.

It has been shown that Notch signalling plays important roles in cutaneous repair [22]. To elucidate the mechanism by which the function of Notch signalling pathway relevant to diabetic wound healing, the expression of the four Notch ligands (Dll-1, Dll-4, Jag1 and Jag2), four Notch receptors (Notch1–4) and Notch target gene *Hes1* in normal or diabetic wound skin tissues were detected. As shown in Figure 2(A), *Jag1*, *Notch1* and *Hes1* mRNA levels were significantly increased in wound skin compared with normal control. We further confirmed that Jag1, Notch1 and Hes1 were up-regulated by Western blot assay (Figure 2B). These results suggested that the increased Jag1 expression in diabetic wound skin tissues is associated with activation of Notch signalling, with an increased expression of Notch target gene *Hes1*.

### Jag1 expression was detected in ESCs

**Figure 3:**
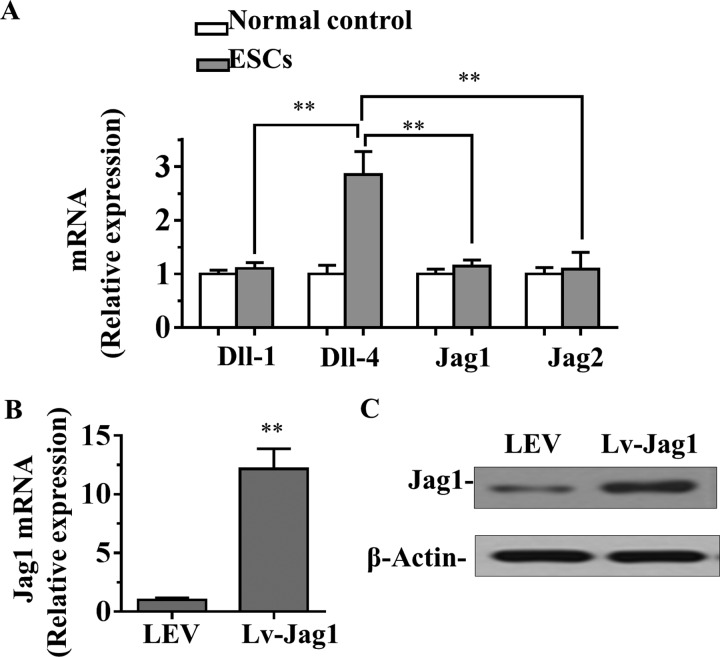
The expression of Jag1 in ESCs (**A**) mRNA level of four Notch ligands (*Dll-1*, *Dll-4*, *Jag1* and *Jag2*) in normal skin cells and ESCs were analysed by qRT-PCR assay. (**B** and **C**) mRNA (B) and protein (C) levels of *Jag1* in Lv-Jag1-ESCs or control lentiviral empty vector ESCs were measured by qRT-PCR and Western blot assay respectively. Data are presented as mean ± S.D. from three independent experiments; **P<0.01 compared with the control group.

If Notch receptor–ligand interactions contribute to the recruitment of ESCs in diabetic wound skin, the Notch ligand should be expressed in the ESCs. Therefore, we detected four Notch ligands (Dll-1, Dll-4, Jag1 and Jag2) in ESCs by qRT-PCR assay. The results showed that the mRNA level of *Jag1* is much higher than Dll-1, Dll-4 and Jag2 (Figure 3A). These data suggested that Jag1 is frequently high expressed in ESCs and likely responsible for the constitutive activation of Notch signalling. Therefore, to further investigate the role of Jag1 in wound healing, the Jag1 overexpression lentiviral vector (Lv-Jag1) and a control lentiviral empty vector was stably transfected into ESCs. The mRNA and protein levels of *Jag1* were significantly increased in Lv-Jag1-ESCs compared with the control LEV group (Figures 3B and 3C). The efficacy of Jag1 overexpressed (Lv-Jag1) ESCs in wound healing was tested in a series of *in vitro* and *in vivo* assays.

### Jag1 promotes ESCs migration *in vitro*

**Figure 4:**
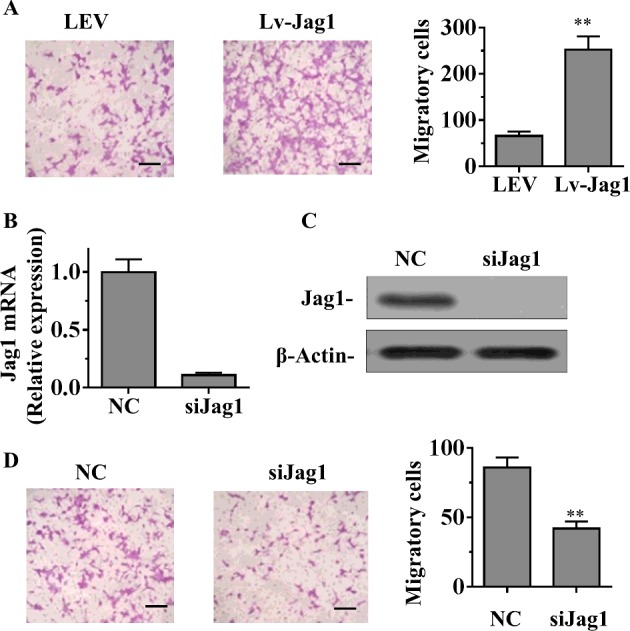
Effect of Jag1 on ESCs migration *in vitro* (**A**) Stably up-regulating Jag1 increased the migration ability of ESCs *in vitro* measured by a transwell chamber. (**B** and **C**) The expression level of *Jag1* was examined by qRT-PCR (B) and Western blot (C) analysis in ESCs treated with siRNAs targeting Jag1. (**D**) Knockdown of Jag1 in ESCs reduced the migration ability. Data are presented as mean ± S.D. from three independent experiments; **P<0.01 compared with the control group; Scale bar, 100 μm.

Given that the expression of Jag1 is closely associated with ESCs, we postulated that Jag1 could have an important role in ESCs migration. To further explore the role of Jag1 in ESCs migration, using a transwell chamber, we determined changes in cell migration after 8 h of incubation. Compared with the LEV cells, Lv-Jag1-ESCs showed significantly increased migratory ability (Figure 4A). Moreover, we suppressed Jag1 with specific siRNA–siJag1. qRT-PCR and Western blot analysis showed that siJag1 was able to effectively knockdown the expression of Jag1 in ESCs cells (Figures 4B and 4C). Further functional studies demonstrated that knockdown of Jag1 significantly decreased the ESCs migration compared with the control group (Figure 4D). Taken together, these results suggested that Jag1 promotes ESCs migration *in vitro*.

### Jag1 accelerates diabetic wound closure *in vivo*

**Figure 5:**
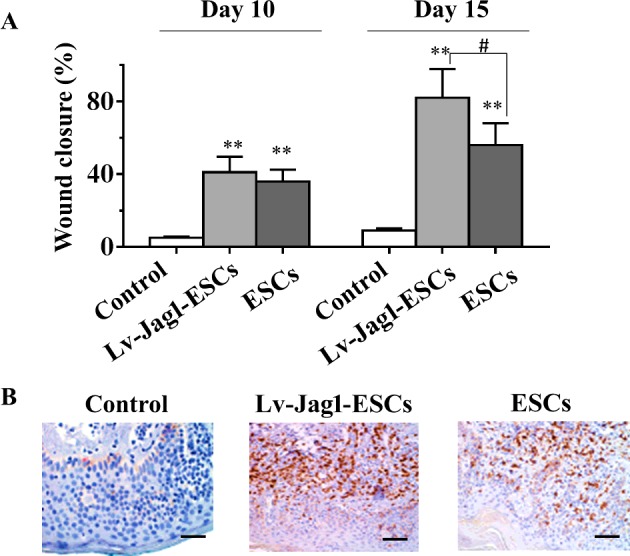
Jag1 accelerated diabetic wound closure (**A**) Topical Lv-Jag1-ESCs, ESCs or PBS control injection in the wound edge for wound healing in the diabetic mice. Comparison of the percentages of the open wound size at day 10 and day 15 between PBS control and Lv-Jag1-ESCs or ESCs-treated diabetic mice. (**B**) Immunohistochemical staining for CD73 in the mouse skin sections after treatment with Lv-Jag1-ESCs, ESCs or PBS control. Five mice per group; Scale bars, 100 μm. Data are presented as the mean ± S.D.;**P<0.01 compared with the PBS control; ^#^*P*<0.05 Lv-Jag1-ESCs compared with ESCs.

To test the hypothesis that overexpression of Jag1 in ESCs could promote diabetic wound healing. First, we made a STZ-induced diabetes mellitus (DM) mouse model, then the diabetic wounds were treated with Lv-Jag1-ESCs, ESCs or PBS control respectively. As shown in Figure 5(A), the difference in wound closure between the ESCs treatment and PBS control group was significantly different at day 10. Moreover, we found that the wound closure of Lv-Jag1-ESCs group compared with ESCs treatment was significantly different at day 15, suggesting Jag1 accelerates wound closure *in vivo.* We further examined the change in ESCs during wound healing by performing immunohistochemical staining of the ESCs markers (CD73) on the skin sections (Figure 5B). Of note, the Lv-Jag1-ESCs treated mice exhibited a significantly increased number of ESCs, as compared with PBS-treated mice. Together, these results indicated that Jag1 increases ESCs migration and improves wound healing in a diabetic mouse model.

## DISCUSSION

Poor wound healing is a major complication in diabetes patients and could result in morbidity or death [23]. Promoting diabetic wound healing by a variety of adult progenitor cells including bone marrow-derived MSCs (BM-MSCs) [24], amniotic MSCs [7] and skin-derived precursor cells (SKPs) [15] have been reported *in vivo*. In the present study, we demonstrated for the first time that topical applications of ESCs enhanced wound healing of diabetic mice. Additionally, we found that overexpression of Jag1 promotes ESCs migration, whereas knockdown Jag1 resulted in a reduction in ESCs migration *in vitro*. Moreover, Jag1 improves diabetic wound healing *in vivo*. Our results provide evidence that ESCs accelerate diabetic wound healing via the Notch signalling pathway.

ESCs have been reported to populate the normal skin niche, remain quiescent and become active after injury, aiding in wound closure [6]. In addition, functional characteristics of ESCs that may benefit wound healing include their ability to migrate to the site of injury or inflammation, participate in regeneration of damaged tissues, stimulate proliferation and differentiation of resident progenitor cells, promote recovery of injured cells through growth factor secretion and matrix remodelling, and exert unique immunomodulatory and anti-inflammatory effects [25,26]. In the present study, a dorsal skin defect in a STZ-induced DM mouse model was used and ESCs were isolated from mouse skin. Our study showed the accelerated healing and closure of diabetic wounds following the local application of ESCs. However, the mechanism of the therapeutic properties of ESCs in the diabetic wound healing is still unclear. Therefore, we further investigated the underlying mechanism of ESCs improving diabetic wound healing.

It is well known that the Notch signalling pathway is critical for cell-fate decisions during development and wound healing [27]. The activation of Notch signalling in keratinocytes is sufficient to cause cell cycle withdrawal and trigger terminal differentiation [28]. It also has been reported that a high level of Notch signalling activity promotes the differentiation of ESCs into keratinocyte and interfollicular lineages [29]. Recently, it is reported that one of Notch ligand Jag1 overexpression in endothelial cells increased vessel density, maturation and perfusion, thus accelerating wound healing [30]. The opposite effect was seen in e*Jag1*cKO animals [30]. Consistent with previous study [30], we confirmed that diabetic wound skin tissues expressed higher level of Jag1 compared with normal mouse skin tissues, and that this was associated with a significant elevated expression of Notch1 and the Notch target gene *Hes1*. These results indicated that Jag1 is likely a major ligand responsible for Notch signalling activation in diabetic wound healing, and that selective targeting of this protein may present a novel therapeutic strategy. It has been reported that Jag1 ablation results in cerebellar granule cell migration defects and depletion of Bergmann glia [31]. In the present study, we showed that Jag1 is high expressed in ESCs and likely responsible for the constitutive activation of Notch signalling. We further demonstrated that overexpression of Jag1 led to an increased cell migration, whereas knockdown of Jag1 decreased the ESCs migration. Previous study showed that the enhanced Notch activity efficiently promoted the corneal wound healing by stimulation of a rapid early cell proliferation [32]. Inhibition of Notch activity in mice significantly delayed the healing of dermal wounds, and activation of Notch activity *in vivo* boosted wound repairing [33]. Consistent with previous report, our *in vivo* study showed that overexpression of Jag1 accelerates wound healing in a diabetic mouse model, suggesting that direct up-regulation of Jag1 may present a novel therapeutic strategy in diabetic wound healing.

In conclusion, this report provides new evidence that ESCs accelerate diabetic wound healing via the Notch signalling pathway. Our results suggest that Jag1 may be a key player of ESCs migration during diabetic wound healing. Therefore, direct targeting Jag1 may present a novel therapeutic strategy in diabetic wound healing.

## Abbreviations

Dll-1: Delta-like1
Dll-4: Delta-like4
DM: diabetes mellitus
ESC: epidermal stem cell
*Hes1*: Hairy Enhancer of Split-1
ICD: intracellular domain
Jag1: Jagged1
Jag2: Jagged2
MSC: mesenchymal stem cell
qRT-PCR: quantitative real-time PCR
STZ: streptozotocin

## References

[1] Ramsey S.D., Newton K., Blough D., McCulloch D.K., Sandhu N., Reiber G.E., Wagner E.H. (1999). Incidence, outcomes, and cost of foot ulcers in patients with diabetes. Diabetes Care.

[2] Singh N., Armstrong D.G., Lipsky B.A. (2005). Preventing foot ulcers in patients with diabetes. JAMA.

[3] Blume P.A., Walters J., Payne W., Ayala J., Lantis J. (2008). Comparison of negative pressure wound therapy using vacuum-assisted closure with advanced moist wound therapy in the treatment of diabetic foot ulcers: a multicenter randomized controlled trial. Diabetes Care.

[4] Veves A., Sheehan P., Pham H.T. (2002). A randomized, controlled trial of Promogran (a collagen/oxidized regenerated cellulose dressing) vs standard treatment in the management of diabetic foot ulcers. Arch. Surg..

[5] Wunderlich R.P., Peters E.J., Lavery L.A. (2000). Systemic hyperbaric oxygen therapy: lower-extremity wound healing and the diabetic foot. Diabetes Care.

[6] Arno A.I., Amini-Nik S., Blit P.H., Al-Shehab M., Belo C., Herer E., Tien C.H., Jeschke M.G. (2014). Human Wharton's jelly mesenchymal stem cells promote skin wound healing through paracrine signaling. Stem Cell Res. Ther..

[7] Kim S.W., Zhang H.Z., Guo L., Kim J.M., Kim M.H. (2012). Amniotic mesenchymal stem cells enhance wound healing in diabetic NOD/SCID mice through high angiogenic and engraftment capabilities. PLoS One.

[8] Lin C.D., Allori A.C., Macklin J.E., Sailon A.M., Tanaka R., Levine J.P., Saadeh P.B., Warren S.M. (2008). Topical lineage-negative progenitor-cell therapy for diabetic wounds. Plast. Reconstr. Surg..

[9] Tark K.C., Hong J.W., Kim Y.S., Hahn S.B., Lee W.J., Lew D.H. (2010). Effects of human cord blood mesenchymal stem cells on cutaneous wound healing in leprdb mice. Ann. Plast. Surg..

[10] Wu Y., Chen L., Scott P.G., Tredget E.E. (2007). Mesenchymal stem cells enhance wound healing through differentiation and angiogenesis. Stem Cells.

[11] Lee M.J., Kim J., Lee K.I., Shin J.M., Chae J.I., Chung H.M. (2011). Enhancement of wound healing by secretory factors of endothelial precursor cells derived from human embryonic stem cells. Cytotherapy.

[12] Kim W.S., Park B.S., Sung J.H., Yang J.M., Park S.B., Kwak S.J., Park J.S. (2007). Wound healing effect of adipose-derived stem cells: a critical role of secretory factors on human dermal fibroblasts. J. Dermatol. Sci..

[13] Shi C., Zhu Y., Su Y., Cheng T. (2006). Stem cells and their applications in skin-cell therapy. Trends Biotechnol..

[14] Toma J.G., McKenzie I.A., Bagli D., Miller F.D. (2005). Isolation and characterization of multipotent skin-derived precursors from human skin. Stem Cells.

[15] Sato H., Ebisawa K., Takanari K., Yagi S., Toriyama K., Yamawaki-Ogata A., Kamei Y. (2015). Skin-derived precursor cells promote wound healing in diabetic mice. Ann. Plast. Surg..

[16] Haniffa M.A., Wang X.N., Holtick U., Rae M., Isaacs J.D., Dickinson A.M., Hilkens C.M., Collin M.P. (2007). Adult human fibroblasts are potent immunoregulatory cells and functionally equivalent to mesenchymal stem cells. J. Immunol..

[17] Riekstina U., Muceniece R., Cakstina I., Muiznieks I., Ancans J. (2008). Characterization of human skin-derived mesenchymal stem cell proliferation rate in different growth conditions. Cytotechnology.

[18] Artavanis-Tsakonas S., Rand M.D., Lake R.J. (1999). Notch signaling: cell fate control and signal integration in development. Science.

[19] Radtke F., Fasnacht N., Macdonald H.R. (2010). Notch signaling in the immune system. Immunity.

[20] Tang Y., Lei X., Jian W., Yan J., Wu Z., Zhao T. (2014). Optimization of streptozotocin dosing for establishing tumor-bearing diabetic mouse models. Nan Fang Yi Ke Da Xue Xue Bao.

[21] Badr G. (2012). Supplementation with undenatured whey protein during diabetes mellitus improves the healing and closure of diabetic wounds through the rescue of functional long-lived wound macrophages. Cell. Physiol. Biochem..

[22] Shi Y., Shu B., Yang R., Xu Y., Xing B., Liu J., Chen L., Qi S., Liu X., Wang P. (2015). Wnt and Notch signaling pathway involved in wound healing by targeting c-Myc and Hes1 separately. Stem Cell Res. Ther..

[23] Kuo Y.R., Wang C.T., Wang F.S., Chiang Y.C., Wang C.J. (2009). Extracorporeal shock-wave therapy enhanced wound healing via increasing topical blood perfusion and tissue regeneration in a rat model of STZ-induced diabetes. Wound Repair Regen..

[24] Kuo Y.R., Wang C.T., Cheng J.T., Wang F.S., Chiang Y.C., Wang C.J. (2011). Bone marrow-derived mesenchymal stem cells enhanced diabetic wound healing through recruitment of tissue regeneration in a rat model of streptozotocin-induced diabetes. Plast. Reconstr. Surg..

[25] Hanson S.E., Bentz M.L., Hematti P. (2010). Mesenchymal stem cell therapy for nonhealing cutaneous wounds. Plast. Reconstr. Surg..

[26] Sasaki M., Abe R., Fujita Y., Ando S., Inokuma D., Shimizu H. (2008). Mesenchymal stem cells are recruited into wounded skin and contribute to wound repair by transdifferentiation into multiple skin cell type. J. Immunol..

[27] Vauclair S., Majo F., Durham A.D., Ghyselinck N.B., Barrandon Y., Radtke F. (2007). Corneal epithelial cell fate is maintained during repair by Notch1 signaling via the regulation of vitamin A metabolism. Dev. Cell.

[28] Rangarajan A., Talora C., Okuyama R., Nicolas M., Mammucari C., Oh H., Aster J.C., Krishna S., Metzger D., Chambon P. (2001). Notch signaling is a direct determinant of keratinocyte growth arrest and entry into differentiation. EMBO J..

[29] Ghadially R. (2012). 25 years of epidermal stem cell research. J. Invest. Dermatol..

[30] Pedrosa A.R., Trindade A., Fernandes A.C., Carvalho C., Gigante J., Tavares A.T., Dieguez-Hurtado R., Yagita H., Adams R.H., Duarte A. (2015). Endothelial Jagged1 antagonizes Dll4 regulation of endothelial branching and promotes vascular maturation downstream of Dll4/Notch1. Arterioscler. Thromb. Vasc. Biol..

[31] Weller M., Krautler N., Mantei N., Suter U., Taylor V. (2006). Jagged1 ablation results in cerebellar granule cell migration defects and depletion of Bergmann glia. Dev. Neurosci..

[32] Lu H., Lu Q., Zheng Y., Li Q. (2012). Notch signaling promotes the corneal epithelium wound healing. Mol. Vis..

[33] Chigurupati S., Arumugam T.V., Son T.G., Lathia J.D., Jameel S., Mughal M.R., Tang S.C., Jo D.G., Camandola S., Giunta M. (2007). Involvement of notch signaling in wound healing. PLoS One.

